# Mechanical, Morphological, Corrosion, and Thermally Activated Dimensional Recovery Behavior of Epoxy Composites Reinforced with Kraft Lignin/Fe–Mn–Si Alloy Hybrid Fillers

**DOI:** 10.3390/polym18131622

**Published:** 2026-06-30

**Authors:** Semih Tanfer Ileri, Mert Yildirim

**Affiliations:** 1 Department of Aeronautical Engineering, Institute of Graduate Studies, Istanbul Gelisim University, 34310 Istanbul, Türkiye; 2 Department of Industrial Engineering, Faculty of Engineering and Architecture, Istanbul Gelisim University, 34310 Istanbul, Türkiye; 3 New Generation Entrepreneurship and Innovation Application and Research Center, Istanbul Gelisim University, 34310 Istanbul, Türkiye

**Keywords:** epoxy composites, kraft lignin, Fe–Mn–Si alloy, SEM analysis, EDS elemental mapping, mechanical properties, corrosion behavior, thermally activated dimensional recovery

## Abstract

In this study, epoxy composites reinforced with kraft lignin, a promising green biofiller, and Fe–Mn–Si alloy particles as metallic functional fillers were developed, and their morphological properties, elemental distribution, mechanical properties, corrosion behavior, and thermally activated dimensional recovery behavior were investigated. Epoxy resin was used as the matrix, while kraft lignin and Fe–Mn–Si particles were incorporated as hybrid fillers. The composites were fabricated by casting with kraft lignin loadings of 1, 3, and 5 wt.% and a fixed Fe–Mn–Si alloy content of 3 wt.%. Neat epoxy was also prepared as a control sample. The specimens were characterized using scanning electron microscopy, energy-dispersive spectroscopy with elemental mapping, tensile testing, Shore D hardness measurements, electrochemical corrosion testing, and dimensional recovery tests. SEM–EDS observations showed that the composite containing 1 wt.% lignin exhibited a relatively uniform fracture morphology and more locally dispersed filler-related elemental signals, whereas higher lignin contents promoted particle-rich regions, microvoid-like features, and increased microstructural heterogeneity. The composite containing 1 wt.% lignin exhibited the highest tensile strength and elongation at break, with values of 39.09 MPa and 2.11%, respectively, and also showed the highest dimensional recovery ratio of 2.5%. The composite containing 3 wt.% lignin exhibited the lowest measured corrosion rate of 0.08 µm/year, while the composite containing 5 wt.% lignin showed the highest elastic modulus and Shore D hardness, with values of 5.80 GPa and 79, respectively. Overall, low lignin loading provided the most balanced mechanical and recovery-related performance, whereas higher lignin contents increased stiffness and hardness but also promoted greater microstructural heterogeneity.

## 1. Introduction

Polymer-based composite materials are increasingly used in engineering applications because of their low density, high specific strength, versatility, design flexibility, and cost-effectiveness [1]. Among polymer matrices, epoxy resins are widely preferred because of their good adhesion, chemical resistance, dimensional stability, and ease of processing. However, neat epoxy systems generally exhibit brittleness, limited crack resistance, and poor functional performance, which may reduce their reliability under mechanical or environmental loading conditions [2]. Therefore, the incorporation of suitable reinforcing phases into epoxy matrices remains an important approach for enhancing mechanical performance, damage tolerance, environmental resistance, and functional behavior [3,4,5].

Smart materials capable of responding to external stimuli have attracted considerable attention for advanced engineering applications [6]. Among these materials, shape memory alloys (SMAs) are functional materials capable of recovering deformation through the shape memory effect, which is associated with a reversible martensitic transformation between austenite and martensite phases [7,8]. Nickel–titanium-based SMAs are the most widely studied systems because of their high recovery strain and excellent functional performance [9,10]. However, their high cost, processing challenges, and sensitivity to compositional variations have encouraged the investigation of alternative SMA systems. Fe–Mn–Si-based alloys have therefore attracted interest as metallic functional materials because of their relatively low cost, favorable mechanical properties, and acceptable recovery-related performance compared with conventional shape memory alloy systems [11,12,13].

In parallel with the development of multifunctional composites, sustainability has become a central consideration in materials design. The use of renewable, low-cost, and bio-derived fillers is consistent with circular-economy principles and the development of more sustainable material systems. Lignin is one of the most abundant natural polymers and is a major by-product of the cellulose pulping process. It has traditionally been treated as a low-value waste material. Nearly 98% of industrially generated lignin is burned for energy recovery, whereas only about 2% is currently utilized in the production of higher-value products, such as specialty chemicals, bio-based polymers, and renewable aromatic compounds [14]. Owing to its aromatic structure, hydroxyl-rich functionality, wide availability, and low cost, lignin has attracted considerable attention as a promising renewable additive for composite materials [15,16,17]. Previous studies on lignin-reinforced polymer systems have mainly focused on mechanical, thermal, rheological, and morphological performance. The reported effects of lignin, however, vary considerably depending on its source, extraction method, loading level, dispersion quality, and interfacial interactions with the polymer matrix [18,19,20,21,22,23,24].

Lignin-reinforced polymer composites and metallic-particle-filled polymer composites have generally been investigated as separate material systems. Consequently, the combined influence of kraft lignin loading on the local microstructure, elemental distribution, mechanical properties, corrosion behavior, and thermally activated dimensional recovery of Fe–Mn–Si-containing epoxy composites remains insufficiently understood.

Addressing this research gap is important because lignin loading may produce competing effects within the hybrid composite system. At low concentrations, lignin may contribute to matrix modification, improved filler dispersion, crack deflection, and energy dissipation. In contrast, excessive lignin loading may promote particle agglomeration, microvoid formation, stress concentration, interfacial defects, and reduced matrix continuity, thereby adversely affecting strength, ductility, corrosion resistance, and recovery-related behavior.

The novelty of the present study lies in the development and comparative evaluation of epoxy composites reinforced with kraft lignin, a promising green biofiller, and Fe–Mn–Si alloy particles as metallic functional fillers. Unlike previous studies that have generally focused on lignin-modified polymers or metallic-particle-reinforced polymer composites separately, the present work evaluates the morphology, local elemental distribution, mechanical properties, electrochemical corrosion behavior, and recovery-related response of a hybrid system containing both fillers within the same epoxy matrix. The Fe–Mn–Si alloy particle content was maintained at 3 wt.%, while the kraft lignin content was systematically varied at 1, 3, and 5 wt.% to determine the influence of lignin loading on fracture morphology, local elemental distribution, tensile properties, hardness, corrosion behavior, and thermally activated dimensional recovery. Neat epoxy was also included as a baseline control to enable formulation-level comparison with the unfilled matrix. Accordingly, this study provides an integrated assessment of the influence of kraft lignin loading on the performance of the kraft lignin/Fe–Mn–Si/epoxy hybrid composite system.

## 2. Materials and Methods

### 2.1. Materials

An epoxy resin was selected as the matrix phase for composite production. The two-component AC-510 epoxy system, consisting of epoxy resin Part A and hardener Part B, was supplied by Armor Chemical (Istanbul, Türkiye).

In composite production, kraft lignin and Fe–Mn–Si alloy particles were used as reinforcement phases. Iron (Fe), manganese (Mn), and silicon (Si) powders used for alloy preparation were supplied by NANOKAR (Istanbul, Türkiye).

### 2.2. Extraction and Preparation of Lignin

Lignin isolation was carried out based on the kraft pulping method, which is widely used in industry. The black liquor was obtained from kraft pulping of pine (*Pinus nigra*) and poplar (*Populus* spp.) under laboratory conditions, and its solid content was adjusted to below 20%. The solutions were heated to approximately 70 °C under continuous stirring using a magnetic stirrer equipped with a hot plate. To precipitate lignin, the pH of the solution was reduced to approximately 2 using a 1 N H_2_SO_4_ solution supplied by Merck KGaA (Darmstadt, Germany). Following acidification, the mixture was maintained at a constant temperature and stirred at 600 rpm for 2 h. It was then centrifuged at 4500 rpm for 10 min to isolate the lignin precipitate. The separated lignin was dried in an oven at 50 °C and subsequently ground into powder form.

### 2.3. Production of Fe–Mn–Si Alloy

The properties of the iron (Fe), manganese (Mn), and silicon (Si) powders used for alloy preparation are summarized below. Chemical Abstracts Service (CAS) numbers, which are unique numerical identifiers assigned to chemical substances, are provided to clearly identify the elemental powders used in this study.

The Fe powder has a Chemical Abstracts Service (CAS) number of 7439-89-6 and a purity of 99.99%. It has an average particle size of 44 µm, a density of 7.87 g/cm^3^, a melting point of 1538 °C, and a boiling point of 2862 °C.

The Mn powder has a CAS number of 7439-96-5 and a purity of 99.99%. It has an average particle size of 44 µm, a density of 7.43 g/cm^3^, a melting point of 1245 °C, and a boiling point of 2150 °C.

The Si powder has a CAS number of 7440-21-3 and a purity of 99.99%. Its particle size is specified as 325 mesh. It has a melting point of 1410 °C and a boiling point of 2355 °C.

In this study, Fe, Mn, and Si elemental powders were mixed in equal weight proportions. Accordingly, the nominal composition of the produced Fe–Mn–Si alloy powder was Fe–33.3Mn–33.3Si wt.%. The produced Fe–Mn–Si alloy powder is described in this study as a metallic functional filler with recovery potential rather than as an optimized commercial Fe–Mn–Si shape memory alloy.

The Fe–Mn–Si alloy was produced using the mechanical alloying technique, a solid-state method that enables homogeneous mixing of elemental powders at the atomic scale. For this purpose, high-energy ball milling was carried out using an MSE-branded ball mill (MSE Technology, Gebze, Kocaeli, Türkiye). This method was preferred because it allows alloying of elements with different melting temperatures, can be performed at room temperature, and contributes to obtaining a more homogeneous microstructure.

Fe, Mn, and Si elemental powders were mixed in equal weight proportions and loaded into a cylindrical metallic milling container. Zirconia balls with a diameter of 6 mm were used as the milling media, with a ball-to-powder mass ratio of 10:1. Mechanical alloying was carried out in a horizontal roller-type ball mill at 400 rpm for 15 h under an inert argon atmosphere.

### 2.4. Composite Formulations

The composite formulations were prepared by varying the kraft lignin content while maintaining the Fe–Mn–Si alloy content at 3 wt.%. Kraft lignin contents of 1, 3, and 5 wt.% were selected to represent low, intermediate, and relatively high loading levels, respectively. These loading levels were selected based on literature reports indicating that low lignin contents may contribute to matrix modification and stress transfer, whereas higher contents may promote agglomeration, brittleness, and microstructural heterogeneity; therefore, the total additive content was kept below 10 wt.%.

The Fe–Mn–Si alloy content was fixed at 3 wt.% to maintain a consistent metallic filler content across the hybrid formulations and to evaluate the influence of lignin loading while limiting excessive particle clustering and disruption of matrix continuity. A lower Fe–Mn–Si content may reduce the metallic functional contribution, whereas a higher Fe–Mn–Si content may promote particle clustering and reduce matrix continuity. A neat epoxy sample without kraft lignin or Fe–Mn–Si particles was also prepared as a baseline control.

The formulations are presented in Table 1.

### 2.5. Production of Composites

Composites were produced using the casting method, which was selected because of its simplicity, cost-effectiveness, and suitability for thermosetting epoxy systems. Compared with techniques such as hot pressing, compression molding, or vacuum infusion, casting does not require high temperatures or complex equipment. In addition, it enables the production of flat composite specimens required for mechanical, corrosion, and recovery tests. Polytetrafluoroethylene (PTFE) sheets with low surface energy were used as the mold material to facilitate easy sample removal.

The mixing conditions were selected based on commonly used processing parameters reported for epoxy-based composite systems to promote homogenization and filler dispersion while limiting excessive air entrapment, temperature rise, and premature viscosity increase [9].

The epoxy resin, Part A, and hardener, Part B, were mixed at a 2:1 weight ratio using a mechanical stirrer at 500 rpm for 5 min. Subsequently, lignin and Fe–Mn–Si alloy powders were added to the epoxy mixture according to the defined composite formulations, and mixing was continued for an additional 5 min to promote filler dispersion. The prepared mixtures were poured into PTFE molds and cured at room temperature for 72 h. After curing, the samples were removed from the molds and cut to dimensions in accordance with the relevant ASTM standards.

### 2.6. Characterization of Composites

#### 2.6.1. Microstructural and Elemental Characterization (SEM–EDS)

The microstructural and elemental characteristics of the composite samples were investigated using scanning electron microscopy coupled with energy dispersive spectroscopy (SEM–EDS). The specimens were examined at an accelerating voltage of 10 kV and a working distance of approximately 10–13 mm. Backscattered-electron (BSE) imaging was used to evaluate the tensile-fracture surface morphology, particle-rich regions, microvoid-like features, possible interfacial gaps, and localized agglomeration.

EDS elemental mapping was performed on selected regions of Groups A, B, and C to examine the local distribution of matrix- and filler-related elements. Elemental maps were obtained for C, Fe, Mn, Si, and other detectable elements, depending on the composition of the analyzed region. The EDS maps were evaluated together with the corresponding BSE images to identify localized filler-related regions and differences in microstructural heterogeneity among the composite groups. Because not all alloying elements were detected in every selected field of view, the EDS results were interpreted as local compositional evidence rather than as confirmation of a uniform Fe–Mn–Si distribution throughout the entire composite.

#### 2.6.2. Tensile Properties

Tensile tests were carried out in accordance with ASTM D3039 [25] using a Devotrans DVT GP D NN universal testing machine (Devotrans, Istanbul, Türkiye). The specimen dimensions were 250 mm × 25 mm × 2.5 mm. The tests were performed at a crosshead speed of 10 mm/min. Three specimens were tested for each experimental group, and the average values were calculated.

#### 2.6.3. Hardness Properties

Hardness measurements were carried out using a Shore D durometer (Loyka LXD2, double-pointer type, M2D; Loyka Instruments, Istanbul, Türkiye) in accordance with ASTM D2240-15 [26]. Measurements were taken at six different locations on each sample surface to minimize local variations, and the average values were reported.

#### 2.6.4. Electrochemical Corrosion Analysis

Electrochemical corrosion analysis were carried out using a three-electrode cell system connected to a Gamry Interface 1000 potentiostat (Gamry Instruments, Warminster, PA, USA). A saturated calomel electrode (SCE) was used as the reference electrode, and a graphite electrode was used as the counter electrode.

The Tafel curves obtained from electrochemical measurements were analyzed to determine the corrosion potential and corrosion current density.

#### 2.6.5. Thermally Activated Dimensional Recovery Test

The thermally activated dimensional recovery behavior of the composites was evaluated by applying a stress of approximately 150 MPa to induce deformation. After unloading, the deformed length of each specimen was measured. The specimens were subsequently heated at 45 °C for 5 min, after which their final lengths were recorded.

The recovery ratio was calculated by comparing the dimensional change after heat treatment with the original length. Specifically, the difference between the final length, Ls, and the initial length, L0, was determined to represent the recovered dimensional change. This value was then divided by the original length and multiplied by 100 to express the recovery ratio as a percentage.

Because the phase constitution and transformation temperatures of the Fe–Mn–Si alloy powder were not verified by X-ray diffraction (XRD) or differential scanning calorimetry (DSC), the measured dimensional change cannot be attributed to a martensite-to-austenite transformation. Therefore, the response is reported only as thermally activated dimensional recovery and is not presented as evidence of a shape memory effect.

## 3. Results and Discussion

### 3.1. Microstructural and Elemental Analysis

The tensile-fracture surfaces of the composite specimens were examined using backscattered-electron scanning electron microscopy (SEM) to evaluate fracture-surface morphology, matrix continuity, particle-rich regions, localized agglomeration, microvoid-like features, matrix cracking, and possible interfacial gaps. EDS elemental mapping was performed to investigate the local distribution of matrix- and filler-related elements.

**Figure 1:**
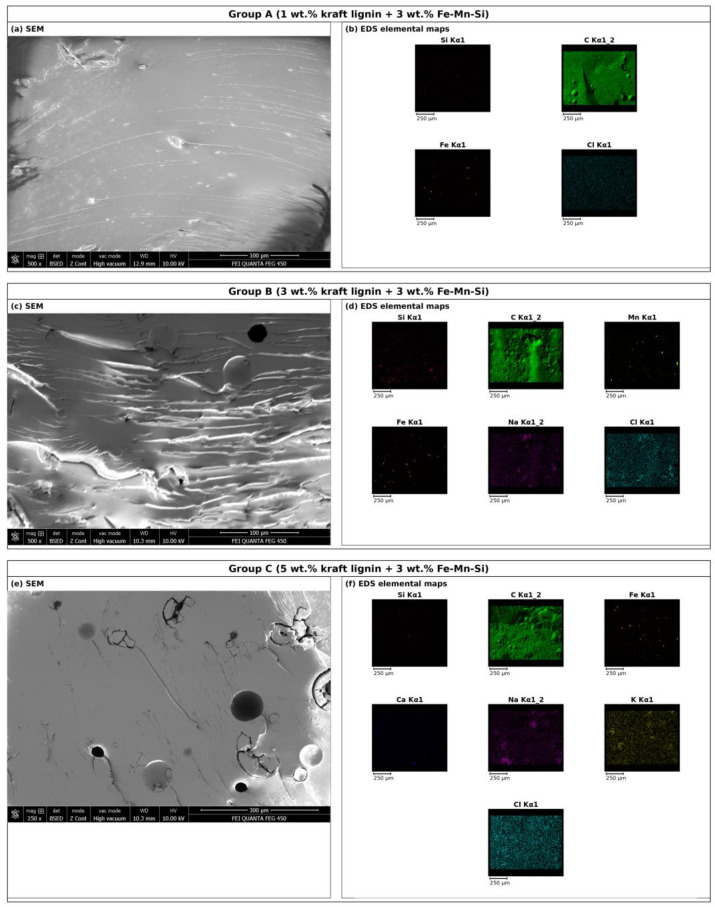
SEM micrographs and corresponding EDS elemental maps of the composites: (**a**,**b**) Group A containing 1 wt.% kraft lignin and 3 wt.% Fe–Mn–Si particles; (**c**,**d**) Group B containing 3 wt.% kraft lignin and 3 wt.% Fe–Mn–Si particles; and (**e**,**f**) Group C containing 5 wt.% kraft lignin and 3 wt.% Fe–Mn–Si particles. Groups A and B were examined at 500× magnification, whereas Group C was examined at 250× magnification. Element labels and scale bars are shown in the individual SEM images and elemental maps.

The SEM micrographs and corresponding EDS elemental maps of Groups A, B, and C are presented in Figure 1.

As shown in Figure 1a, Group A exhibited a comparatively smooth and continuous fracture-surface morphology. Matrix-deformation lines and crack-deflection traces were visible across the examined region, suggesting that the crack path was locally altered during tensile loading. Extensive particle pull-out, severe microvoid formation, and pronounced interfacial gaps were not dominant within the selected field of view. This morphology suggests that the low lignin loading limited the formation of severe local defects and maintained comparatively good matrix continuity. These observations are qualitatively consistent with the higher tensile strength and elongation at break measured for Group A compared with the neat epoxy control and the other hybrid formulations.

The corresponding EDS elemental maps of Group A are shown in Figure 1b. Carbon was detected throughout the analyzed region, consistent with the epoxy- and lignin-rich matrix. Fe and Si were observed as sparse and localized signals within the carbon-rich phase, and no large continuous Fe- or Si-enriched regions were identified in the selected area. This local elemental distribution is consistent with the comparatively uniform fracture morphology observed in Figure 1a. A weak and broadly distributed Cl signal was also detected, whereas Mn was not detected within the selected field of view. Because EDS mapping represents only a limited analysis area, these results should be interpreted as local evidence of Fe- and Si-containing regions rather than as confirmation of a homogeneous distribution of all Fe–Mn–Si constituents throughout the entire composite.

Compared with Group A, Group B exhibited a rougher and more heterogeneous fracture surface, as shown in Figure 1c. Pronounced matrix-deformation features, localized particle-rich regions, and microvoid-like cavities were observed. Increasing the lignin content to 3 wt.% therefore appeared to increase local microstructural heterogeneity within the epoxy matrix. The particle-rich and microvoid-like regions may have acted as local stress-concentration sites during tensile loading, thereby facilitating crack initiation and reducing effective stress transfer. This interpretation is consistent with the lower tensile strength and elongation at break measured for Group B compared with Group A.

The corresponding EDS maps of Group B are presented in Figure 1d. Carbon was distributed throughout the analyzed area, while Fe, Mn, and Si were detected as discrete and localized signals within the carbon-rich matrix. Several element-enriched regions were observed, indicating a heterogeneous local distribution of the detectable metallic filler-related elements. This observation agrees qualitatively with the rougher and more heterogeneous fracture morphology observed in Figure 1c. Na was also detected and may be associated with residual inorganic constituents originating from the kraft lignin source or its preparation process. A weak and broadly distributed Cl signal was also observed. As the EDS results represent only the selected field of view, they were interpreted as local compositional evidence rather than as confirmation of the elemental distribution throughout the bulk composite.

The fracture surface of Group C is shown in Figure 1e. Compared with Groups A and B, Group C exhibited more pronounced microstructural heterogeneity, including particle-rich regions, localized agglomeration, microvoid-like cavities, and crack-like gaps around some particles. These features may indicate local interfacial separation or particle debonding during tensile fracture. Such heterogeneous regions can promote stress concentration and facilitate crack initiation and propagation. Although the higher lignin content contributed to increased elastic modulus and hardness, the observed agglomerated regions, microvoid-like features, and possible interfacial gaps may have reduced effective stress transfer and limited ductility. These observations are qualitatively consistent with the lower tensile strength and elongation at break of Group C compared with Group A. However, because the Group C micrograph was obtained at a lower magnification than those of Groups A and B, direct quantitative comparison of individual feature sizes was not performed.

The corresponding EDS elemental maps of Group C are presented in Figure 1f. Carbon was detected throughout the analyzed region, consistent with the epoxy- and lignin-rich matrix. Fe and Si were observed as discrete and localized signals, with several locally concentrated Fe-rich regions. These features are consistent with the particle-rich areas and heterogeneous fracture morphology observed in Figure 1e. Na, K, and Ca were also detected and may originate from residual inorganic constituents associated with the kraft lignin source or processing route. A weak and broadly distributed Cl signal was also observed, whereas Mn was not detected within the selected field of view. Because EDS mapping covers only a limited analysis area, these findings were interpreted as local compositional evidence rather than as confirmation of the spatial distribution of all Fe–Mn–Si constituents throughout the entire composite.

Overall, the combined SEM and EDS observations indicate that the local microstructural heterogeneity increased with increasing kraft lignin content. Group A exhibited the most continuous fracture morphology and fewer pronounced particle-rich or microvoid-like regions, whereas Groups B and C showed greater local heterogeneity. These morphological differences are qualitatively consistent with the mechanical-property results. The comparatively uniform morphology of Group A may have supported more effective stress transfer and delayed premature crack initiation, while the heterogeneous regions observed in Groups B and C may have promoted local stress concentration. Nevertheless, these relationships should be regarded as morphology-based interpretations rather than as definitive evidence of the mechanisms governing the measured mechanical behavior.

### 3.2. Mechanical Properties

**Figure 2:**
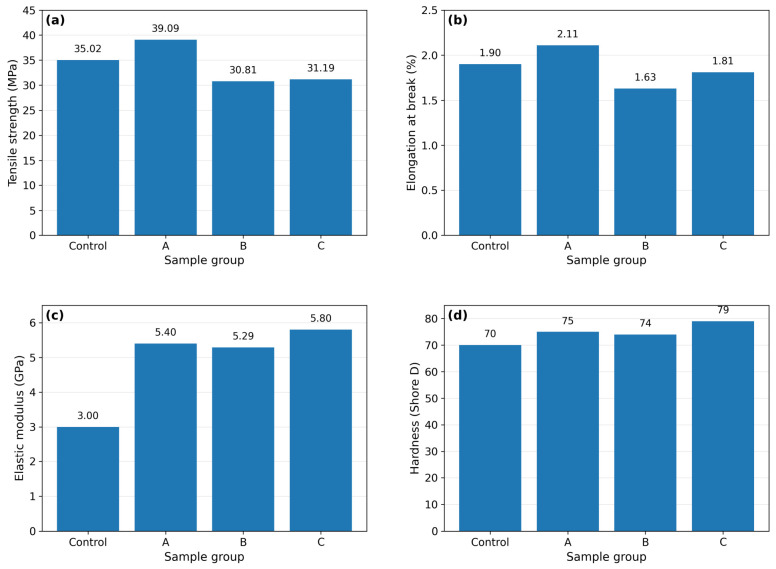
Mechanical properties of neat epoxy and the kraft lignin/Fe–Mn–Si-reinforced composites: (**a**) tensile strength, (**b**) elongation at break, (**c**) elastic modulus, and (**d**) Shore D hardness. Control represents neat epoxy, whereas Groups A, B, and C contain 1, 3, and 5 wt.% kraft lignin, respectively, together with a fixed Fe–Mn–Si particle content of 3 wt.%. Values represent the mean of three specimens.

The mechanical properties of neat epoxy and the kraft lignin/Fe–Mn–Si-reinforced composites are presented numerically in Table 2 and Table 3 and graphically in Figure 2. The neat epoxy sample was included as a control to establish a baseline for evaluating the overall influence of the hybrid fillers on the mechanical performance of the composites.

As shown in Table 2 and Table 3 and Figure 2, the mechanical response of the kraft lignin/Fe–Mn–Si-reinforced composites varied with kraft lignin loading. Group A exhibited the highest tensile strength and elongation at break, whereas Group C exhibited the highest elastic modulus and Shore D hardness.

**Figure 3:**
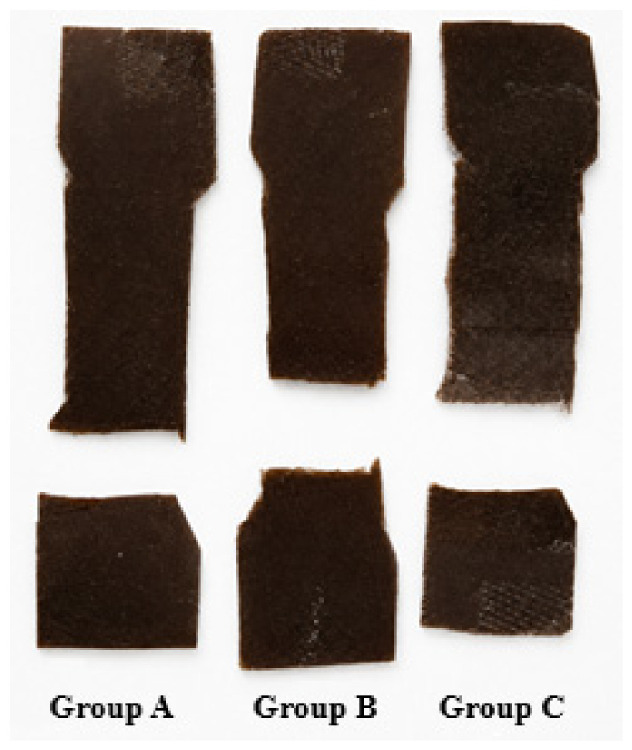
Photographs of tensile-fractured composite specimens after tensile testing. Groups A, B, and C represent epoxy composites containing 1, 3, and 5 wt.% kraft lignin, respectively, together with a fixed Fe–Mn–Si alloy particle content of 3 wt.%.

Figure 3 presents photographs of the tensile-fractured composite specimens after testing.

The fracture locations were visually examined, and the specimens predominantly failed within the gauge section rather than near the grip region. This observation indicates that the measured tensile properties were not primarily governed by grip-induced failure.

As shown in Table 2 and Figure 2a,b, the neat epoxy control exhibited a tensile strength of 35.02 MPa and an elongation at break of 1.90%. Group A, containing 1 wt.% kraft lignin and 3 wt.% Fe–Mn–Si particles, exhibited the highest tensile strength and elongation at break among all investigated formulations, with values of 39.09 MPa and 2.11%, respectively. These values represent increases of approximately 11.6% in tensile strength and 11.1% in elongation at break compared with the neat epoxy control. Thus, the low lignin loading used in Group A improved both the strength and deformation capability of the epoxy-based hybrid composite under the applied test conditions.

The improved tensile properties of Group A may be associated with its comparatively continuous fracture morphology and the absence of large continuous Fe- or Si-rich regions within the selected EDS field of view. At low lignin loading, the comparatively well-distributed filler-containing regions may support stress transfer, crack deflection, and energy dissipation without producing extensive particle-rich regions or pronounced microstructural defects. In addition, the hydroxyl-rich structure of kraft lignin may promote physical interactions, including hydrogen bonding, with the epoxy network. These combined effects may have enhanced the ability of the composite to sustain tensile loading and undergo deformation before failure.

When the lignin content was increased to 3 wt.%, Group B exhibited a tensile strength of 30.81 MPa and an elongation at break of 1.63%. These values were lower than those of both the neat epoxy control and Group A. Compared with Group A, the tensile strength and elongation at break decreased by approximately 21.2% and 22.7%, respectively. This reduction may be associated with increased particle–particle interactions, localized lignin-rich or particle-rich regions, and greater microstructural heterogeneity. Such regions may act as local stress-concentration sites, facilitate microcrack initiation, and reduce effective stress transfer and ductility.

Group C, containing 5 wt.% kraft lignin and 3 wt.% Fe–Mn–Si particles, exhibited a tensile strength of 31.19 MPa and an elongation at break of 1.81%. These values were slightly higher than those of Group B but remained lower than those of the neat epoxy control and Group A. The results indicate that increasing the lignin content beyond 1 wt.% did not provide further improvement in tensile performance. Although higher lignin loading may contribute to increased stiffness, it may also promote local agglomeration, microvoid-like features, and reduced matrix continuity, thereby limiting effective stress transfer between the matrix and filler phases.

As shown in Table 3 and Figure 2c,d, the neat epoxy control exhibited an elastic modulus of 3.00 GPa and a Shore D hardness of 70. All composite groups exhibited higher elastic modulus values than neat epoxy. Group A showed an elastic modulus of 5.40 GPa, while Groups B and C exhibited values of 5.29 and 5.80 GPa, respectively. The highest elastic modulus was obtained for Group C, corresponding to an increase of approximately 93.3% compared with the neat epoxy control. This result indicates that the highest lignin loading produced the stiffest formulation among the investigated groups.

The Shore D hardness values followed a generally similar trend. Group A exhibited a hardness of 75, Group B exhibited a hardness of 74, and Group C exhibited the highest hardness value of 79. Compared with the neat epoxy control, the hardness of Group C increased by approximately 12.9%. The increase in elastic modulus and hardness at higher lignin loading may be related to the greater solid filler content and increased restriction of polymer-chain mobility. However, the increased stiffness and hardness did not result in improved tensile strength or elongation at break.

Overall, the mechanical results demonstrate that the effect of kraft lignin loading depends on the balance among filler content, local filler distribution, matrix continuity, and defect formation. The low lignin loading used in Group A provided the most favorable combination of tensile strength and elongation at break. In contrast, increasing the lignin content enhanced stiffness and hardness but reduced tensile strength and ductility relative to Group A. The SEM and EDS observations suggest that the particle-rich regions, microvoid-like features, and greater local microstructural heterogeneity observed in Groups B and C may have promoted stress concentration and less effective stress transfer. Nevertheless, these relationships should be regarded as morphology-based interpretations rather than definitive evidence of the mechanisms governing the measured mechanical behavior.

These trends are consistent with previous studies reporting that low lignin contents may support matrix modification, crack deflection, stiffness enhancement, and energy dissipation, whereas higher lignin loadings may promote particle agglomeration, stress concentration, interfacial defects, and reduced ductility [27,28,29,30]. The mechanical response of lignin-containing polymer composites is also known to depend strongly on lignin source, particle size, surface chemistry, matrix compatibility, and processing conditions.

### 3.3. Corrosion Behavior

The corrosion rates of neat epoxy and the lignin/Fe–Mn–Si hybrid composites, expressed as annual thickness loss (µm/year), are presented in Table 4.

The neat epoxy control exhibited the highest measured corrosion rate at 0.15 µm/year. All hybrid formulations showed lower measured corrosion rates than neat epoxy under the applied test conditions. Because single-filler control groups were not included, these differences were interpreted as the overall responses of the hybrid formulations rather than as effects attributable independently to kraft lignin or Fe–Mn–Si particles.

Among the composites, Group B exhibited the lowest measured corrosion rate, at 0.08 µm/year. This value was approximately 46.7% lower than that of neat epoxy and 27.3% lower than that of Group A. The comparatively low corrosion rate of Group B may reflect a favorable balance between increased diffusion-path tortuosity and limited defect formation within the composite structure.

Group A exhibited a corrosion rate of 0.11 µm/year, which was lower than that of neat epoxy but higher than that of Group B. Thus, the Group A formulation showed a lower measured corrosion rate than the unfilled matrix under the applied conditions, although it did not provide the most favorable corrosion response among the investigated hybrid formulations.

Group C showed a corrosion rate of 0.10 µm/year, which was slightly higher than that of Group B but remained lower than that of neat epoxy. The increase from Group B to Group C indicates that increasing the lignin content beyond 3 wt.% did not lead to a further reduction in the measured corrosion rate. The particle-rich regions, microvoid-like features, and local microstructural heterogeneity observed in Group C may have provided preferential pathways for electrolyte ingress, thereby reducing its barrier efficiency. Nevertheless, this explanation should be regarded as a morphology-based hypothesis rather than as a confirmed electrochemical mechanism.

Overall, all investigated hybrid formulations exhibited lower measured corrosion rates than neat epoxy under the applied test conditions, with Group B showing the lowest value. Within the investigated formulation range, the composite containing 3 wt.% kraft lignin and 3 wt.% Fe–Mn–Si particles exhibited the most favorable corrosion behavior.

### 3.4. Thermally Activated Dimensional Recovery

The thermally activated dimensional recovery values of neat epoxy and the lignin/Fe–Mn–Si hybrid composites are presented in Table 5.

The comparatively higher recovery observed for Group A may be associated with its relatively uniform fracture morphology and the absence of large continuous Fe- or Si-rich regions within the selected EDS field of view. This local microstructural condition may have reduced constraints on matrix deformation and facilitated dimensional recovery during heating. However, because an epoxy/Fe–Mn–Si single-filler control was not included, the specific contribution of the metallic filler could not be distinguished from other possible effects, including polymer-matrix relaxation, residual-stress release, matrix stiffness, and local filler–matrix constraints.

Group B showed the lowest measured recovery ratio among the hybrid formulations. Its rougher and more heterogeneous fracture morphology, together with localized Fe-, Mn-, and Si-containing regions, may have restricted matrix mobility and limited dimensional recovery. Group C exhibited a higher recovery value than Group B but remained below Group A. The greater stiffness of Group C may have contributed to elastic restoring forces during heating; however, the particle-rich regions, microvoid-like features, and possible local interfacial weakening observed by SEM may have restricted more extensive recovery.

Overall, the dimensional recovery response varied with lignin loading within the investigated hybrid composite system. The observed dimensional changes may reflect the combined effects of polymer-matrix relaxation, residual-stress release, matrix stiffness, thermal expansion and contraction, and local filler–matrix constraints. Because the phase constitution and transformation temperatures of the mechanically alloyed Fe–Mn–Si powder were not verified by XRD or DSC, the measured response was not attributed to a martensite-to-austenite transformation. Accordingly, the results are discussed only in terms of thermally activated dimensional recovery and not as evidence of a shape memory effect.

### 3.5. Proposed Mechanism

**Figure 4:**
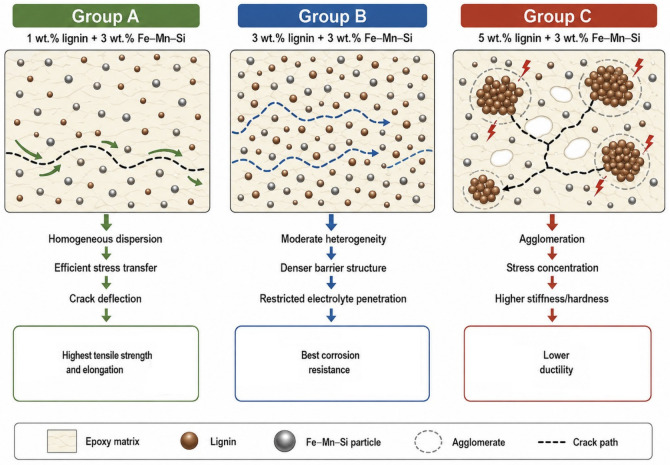
Proposed mechanism illustrating the effect of lignin loading on filler dispersion, fracture behavior, corrosion response, and mechanical performance of epoxy/Fe–Mn–Si hybrid composites. The green dashed arrow indicates crack deflection, the blue dashed arrows indicate restricted electrolyte penetration, and the black dashed arrow indicates the crack path. The red lightning symbols represent local stress-concentration regions, while the downward solid arrows show the proposed cause–effect relationship between microstructural features and composite performance. Particle-rich regions, microvoid-like defects, and possible interfacial gaps represent local heterogeneities that may become more pronounced at higher lignin loadings.

Figure 4 presents the proposed mechanism explaining the role of lignin loading in the epoxy/Fe–Mn–Si hybrid composite system.

At low lignin content, Group A showed a relatively uniform fracture morphology and no large continuous Fe- or Si-rich regions within the selected EDS mapping field. This local microstructural condition may promote stress transfer, crack deflection, and energy dissipation, contributing to improved tensile strength and elongation. At moderate lignin content, Group B exhibited localized element-enriched regions and a more heterogeneous fracture morphology, while still providing the lowest measured corrosion rate. At the highest lignin content, Group C showed more pronounced particle-rich regions, microvoid-like features, and local heterogeneity, which may promote stress concentration and reduce effective load transfer.

Metallic particles embedded in polymer matrices may influence composite behavior through stress transfer, stiffness enhancement, local deformation constraints, and thermal-stress redistribution. In the present study, the measured dimensional changes are interpreted as the overall response of the hybrid epoxy/lignin/Fe–Mn–Si formulations and are not assigned to a martensitic transformation.

This explanation is consistent with previous studies on polymer composites reinforced with functional fillers [31,32].

## 4. Conclusions

This study presents the development of hybrid epoxy composites reinforced with kraft lignin, a promising green biomaterial, and Fe–Mn–Si alloy particles, and evaluates their synergistic effect. By systematically varying the kraft lignin content while keeping the Fe–Mn–Si alloy loading constant, the morphological, elemental distribution, mechanical properties, corrosion behavior, and thermally activated dimensional recovery behavior of the composites were investigated.

The composite containing 1 wt.% lignin exhibited the most balanced performance, achieving the highest tensile strength (39.09 MPa), elongation at break (2.11%), and dimensional recovery ratio (2.5%), together with a comparatively uniform fracture morphology. Increasing the lignin content enhanced stiffness and hardness but promoted greater microstructural heterogeneity. The composite containing 3 wt.% lignin showed the lowest corrosion rate (0.08 µm/year), whereas the composite containing 5 wt.% lignin exhibited the highest elastic modulus (5.80 GPa) and Shore D hardness (79). These findings indicate that lignin loading can be adjusted to tailor the performance of kraft lignin/Fe–Mn–Si/epoxy-reinforced composites according to the targeted property.

Potential application areas for the epoxy composites reinforced with kraft lignin/Fe–Mn–Si alloy hybrid fillers include non-structural automotive interior components, aircraft interior parts, and lightweight panels. However, further mechanical, thermal, and environmental validation is required before practical implementation.

Future studies should include XRD and DSC analyses to clarify the phase structure and transformation behavior of the Fe–Mn–Si particles. Electrochemical impedance spectroscopy and post-corrosion surface analyses are also recommended to validate the proposed barrier mechanisms, while FTIR or XPS analyses may help identify possible interfacial interactions between kraft lignin and the epoxy matrix.

## Figures and Tables

**Table 1:** Formulations of neat epoxy and composite samples.

Sample Group	Lignin (%)	Fe–Mn–Si Alloy (%)
Control	0	0
A	1	3
B	3	3
C	5	3

**Table 2:** Tensile strength (MPa) and elongation at break (%) values of neat epoxy and composite samples.

Sample Group	Tensile Strength (MPa)	Elongation at Break (%)
Control	35.02	1.90
A	39.09	2.11
B	30.81	1.63
C	31.19	1.81

**Table 3:** Elastic modulus (GPa) and hardness values of neat epoxy and composite samples.

Sample Group	Elastic Modulus (GPa)	Hardness (Shore D)
Control	3.00	70
A	5.40	75
B	5.29	74
C	5.80	79

**Table 4:** Corrosion rate values of neat epoxy and composite samples.

Sample Group	Corrosion Rate (µm/Year)
Control	0.15
A	0.11
B	0.08
C	0.10

**Table 5:** Percentage recovery to the original length of neat epoxy and composite samples after deformation.

Sample Group	Recovery (%)
Control	0
A	2.5
B	1.5
C	2

## Data Availability

The raw data supporting the conclusions of this article will be made available by the authors on request.
